# Community perceptions towards children living with albinism in Africa: An integrative review

**DOI:** 10.4102/ajod.v14i0.1718

**Published:** 2025-10-31

**Authors:** Tumisho Mokwele, Ramadimetja Shirley Mooa, Nombulelo V. Sepeng

**Affiliations:** 1 Department of Nursing, Faculty of Health Sciences, University of Pretoria, Pretoria, South Africa

**Keywords:** albinism, children, culture, community, perception

## Abstract

**Background:**

Community perceptions of children with albinism often influence their inclusion, safety and access to education, violating their basic human rights. Children with albinism in Africa encounter social challenges because of myths, superstitions and discrimination because of a lack of knowledge about albinism, making it difficult for the mother who has given birth to a child with albinism.

**Objectives:**

To synthesise the existing literature on community perceptions of children with albinism in Africa.

**Method:**

An integrative review process was used, which involved five steps to review the literature: problem identification, literature search and data collection, data evaluation, data analysis and presentation of findings. Electronic searches were performed in multiple databases, including EBSCOhost, PubMed, Scopus, Web of Science, Google Scholar, Proquest and grey literature.

**Results:**

Community perceptions of children with albinism are embedded within their cultural beliefs and spirituality. Children with albinism continue to be alienated in their communities as their existence is associated with shame, judgement from God or ancestors and superpowers.

**Conclusion:**

Awareness campaigns should be continuous in communities to dispel the myths surrounding albinism. This can be achieved by involving community leaders, religious organisations and organisations that advocate for people with albinism to ensure that our communities create a safe environment for these children.

**Contribution:**

This review may help understand perceptions of albinism and may assist in developing community-based interventions to support caregivers of children with albinism.

## Introduction

Albinism is a genetic condition characterised by a lack of melanin production, resulting in distinctive features such as light skin, hair and eyes, which may cause visual impairment (Kromberg & Manga 2018). While the biological aspects of albinism are well documented, societal perceptions of the condition differ, as they are widely influenced by culture. Cultural perceptions of albinism may differ depending on historical, social, religious, cultural and environmental factors (Kajuri & Nyimbi 2020).

The prevalence of people living with albinism is estimated to be 1:17 000 worldwide, with sub-Saharan Africa accounting for 15 000 and South Africa accounting for 1:3900 (Kromberg & Kerr 2022). In 2006, the United Nations played a significant role in adopting the Convention on the Rights of Persons with Disabilities (CRPD). The CRPD aims to protect and ensure that people with disabilities enjoy equal human rights and freedom (United Nations 2008). Although people living with albinism are a minority in South Africa, the constitution has ensured that their rights are not excluded; therefore, there is legislation for non-discrimination against people with disabilities (Mswela 2018). Mswela (2018) further noted that legislation such as the *Promotion of Equality and Prevention of Unfair Discrimination Act, Employment Equity Act 19* and *Labour Relations Act* ensure that laws for people with disabilities are implemented and protected.

The recorded history of albinism (Hilton 2021) was confined to myths (Bradbury-Jones et al. 2018; Kromberg & Manga 2018) and beliefs, which either saw people living with albinism as a curse or a result of disrespecting the ancestors and therefore seen as a punishment (Taylor et al. 2021). As a result, people living with albinism are still seen as subhuman, with their rights disregarded and mistreated (Bradbury-Jones et al. 2018).

Dapi, Tambe and Monebenimp (2018) emphasised in their work that people with albinism are prone to experiencing attacks and prejudice. There are harmful myths that suggest that sleeping with a person with albinism can bring them luck and that mutilating them can make one rich (Daklo & Obadire 2024). These myths and attacks, according to Masanja, Imori and Kaudunde (2020a), stem from a lack of knowledge and negative attitudes towards albinism among people with lower literacy levels. However, other gruesome acts recorded towards people living with albinism have been committed by educated people, as was the case in Witbank, South Africa, in 2018, where the perpetrator was a schoolteacher (Njilo 2019).

African countries, through the African Union, adopted the Regional Action Plan in 2019 to reduce brutality against people with albinism and promote inclusivity (Ero et al. 2021). According to the Legal and Human Rights report (2019), in Tanzania, the Regional Action Plan has enabled countries to put the spotlight on the challenges people with albinism face and therefore enabled them to establish stricter laws against perpetrators who attack people with albinism. This Regional Action Plan has been adopted in South Africa, leading to increased awareness and advocacy; however, more can still be done to ensure the safety and security of people with albinism. Several studies have investigated the challenges and experiences of people with albinism in Africa; however, limited research has focused on the perceptions of community members in Africa. Therefore, this review seeks to synthesise the available literature regarding community members’ perceptions towards children with albinism in Africa by searching published and grey literature.

## Methods

**FIGURE 1:**
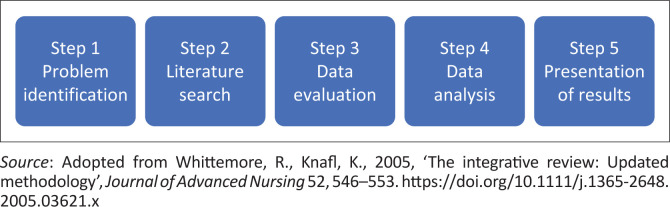
Integrative review process.

An integrative review is a comprehensive methodological approach that combines data from theoretical and empirical literature (Souza, Silva & Carvalho 2010:103) to determine what is known about the subject matter to generate a new perspective and framework (Russell 2005). This review utilised the Whittemore and Knafl (2005) approach, which uses five steps to review the literature: problem identification, literature search and data collection, data evaluation, data analysis and presentation of findings. Figure 1 illustrates the steps followed in this study.

### Step 1: Problem identification

Caregivers of children with albinism frequently experience societal stigmatisation following the birth of their child with albinism (Baker et al. 2021). They are accused by those around them of having extramarital affairs or of sleeping with a white man (Ero et al. 2021). This may result in the caregiver being abandoned by their partner or being divorced, as the partner does not want to bear the stigma of having a child with albinism, causing the caregiver to raise the child alone (Kajuri & Nyimbi 2020). According to Steyn (2022), caregivers are left with only the choice of abandoning or killing their children to avoid being victimised by the family and the community. Consequently, those who choose to raise children with albinism often encounter challenges because of inadequate community support and exclusion (Owoeye et al. 2023).

The research question was formulated using the adapted population, interest and outcome (PIO) framework (Methley et al. 2014):

- P – Population: the population include community teachers, relatives, and mothers.
- I – Interest: the interest involves perceptions, attitudes, and knowledge.
- O – Outcome: the outcome refers to perceptions of the community towards a child with albinism.

Therefore, the following review question was formulated: What is the available information in the literature about the perceptions of a community towards children with albinism in Africa from July 2013 to August 2024? This period was selected as the first author anticipated obtaining recent and relevant reports and studies related to the topic.

### Step 2: Literature search

The literature search is a fundamental step in an integrative literature review to ensure that the search is performed thoroughly and comprehensively (Whittemore & Knafl 2005). The search was conducted to identify, analyse and synthesise results from primary studies to determine the information available on the perceptions of a community towards children with albinism. The literature search was conducted by the first author with the assistance of the librarian responsible for the Health Sciences Faculty between 2023 and 2024. The electronic databases searched were Cochrane, Scopus, PubMed, EBSCOhost, Medline, Google Scholar and government and non-governmental organisations’ websites to ensure that a comprehensive search was conducted.

The following mesh terms were used to search for literature on electronic databases: (‘Albinism’ OR ‘Albino’ AND ‘Community’ OR ‘Family’ OR ‘Relatives’ OR ‘Neighbours’ OR ‘Children’) AND (‘Perception’ OR ‘Knowledge’ OR ‘Attitude’) AND (Africa OR Angola OR Benin OR Botswana OR ‘Burkina Faso’ OR Burundi OR ‘Cabo Verde’ OR Cameroon OR ‘Central African Republic’ OR Chad OR Comoros OR Congo OR ‘Cote d’ivoire’ OR ‘Equatorial Guinea’ OR Eritrea OR Eswatini OR Ethiopia OR Gabon OR ‘Gambia, the’ OR Ghana OR Guinea OR ‘Guinea-Bissau’ OR Kenya OR Lesotho OR Liberia OR Madagascar OR Malawi OR Mali OR Mauritania OR Mauritius OR Mozambique OR Namibia OR Niger OR Nigeria OR Rwanda OR ‘Sao tome and Principe’ OR Senegal OR Seychelles OR ‘Sierra Leone’ OR Somalia OR ‘South Africa’ OR Sudan OR Tanzania OR Togo OR Uganda OR Zambia OR Zimbabwe OR Morocco OR Algeria OR Tunisia OR Libya OR Egypt OR ‘Western Sahara’). The Boolean operators ‘AND’ and ‘OR’ were used to combine the concepts.

The electronic search yielded 338 published studies, seven citation sources and five grey literature items from non-governmental organisations. These studies were then transferred to Covidence. Duplicates were removed using Covidence software, and 190 studies were screened, of which 42 were eligible; however, 33 studies were excluded because of inappropriate study design, population and setting. Therefore, nine studies were included in this review.

**FIGURE 2:**
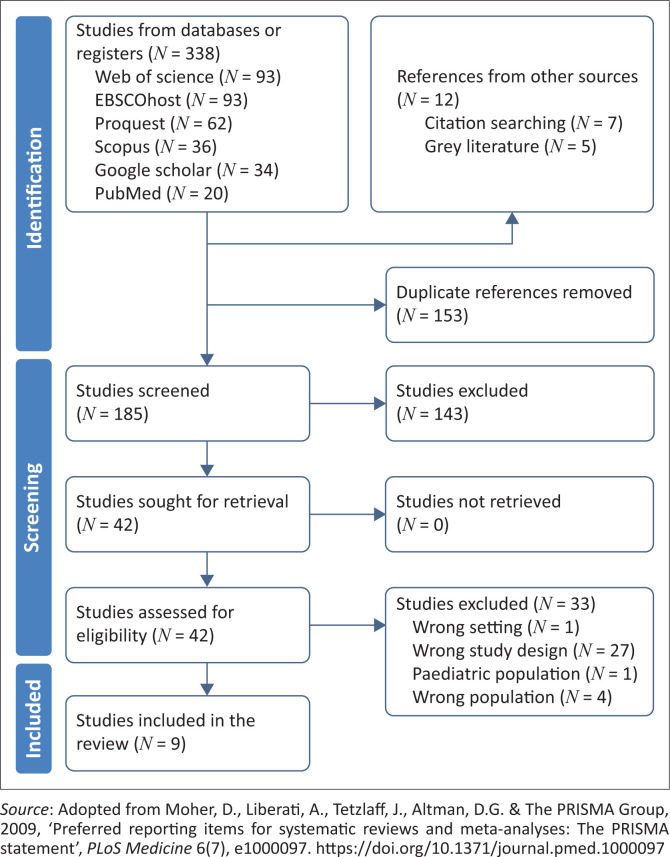
Preferred Reporting for Systematic Reviews and Meta-analysis flow diagram.

The first author and the second author completed the screening process to identify relevant studies. This process was performed using the Preferred Reporting for Systematic Reviews and Meta-analysis table (PRISMA), as illustrated in Figure 2.

### Inclusion and exclusion criteria

The review included primary studies, secondary studies, qualitative, quantitative and mixed-methods design as well as grey literature published from 2013 to 2024. The population had to be a community with concepts related to their perceptions, attitudes and responses towards children with albinism. The studies had to be conducted in Africa and written in English.

### Step 3: Data evaluation

The data evaluation stage includes critically assessing the quality of the primary and grey literature selected to evaluate their strengths and weaknesses (Whittemore & Knafl 2005). The quality of the data was evaluated using a 10-question Critical Appraisal Skills Programme (CASP 2018) for qualitative and quantitative research. The following criteria were used to critically appraise research papers: having a clear aim, appropriate methodology, appropriate research design, clear data collection process, ethical considerations, applied rigorous data analysis and clear findings, limitations and recommendations.

The Mixed-Methods Appraisal Tool (MMAT 2018) was used for mixed-methods studies. The following criteria were used to assess mixed-methods studies: a clear rationale for employing mixed-methods, integration of different components, adequately interpreted qualitative and quantitative components, divergences and inconsistencies between qualitative and quantitative results addressed and the components of the studies adhering to the quality criteria of the methods involved.

### Step 4: Data analysis

The data extraction format used was adapted from Souza et al.’s (2010) data extraction table. The table indicates the characteristics of the studies eligible for extraction, consisting of the author’s name, year and place where the study was conducted. It also includes the aim of the study, research methodology, population, data collection methods and findings (Table 1). Nine studies were eligible for inclusion and were extracted. The characteristics of the study are presented in Table 1.

**TABLE 1:** Data extraction table.

No.	Author(s), year, place	Aim of the study	Research methodology	Population	Data collection method	Findings
1	Bradbury et al. (2018) Uganda	‘To explore the beliefs that surround people with albinism’.	Qualitative design	People with albinism, parents of children with albinism and people with expert and professional insight regarding the issue.	Group discussion Individual interviews	Mothers who gave birth to a child with albinism were accused of being impregnated by a ghost. School children thought that albinism might be contagious. Teachers thought that children with albinism should be educated separately.
2	Likumbo, De Villiers and Kyriacos (2021) Malawi	‘To explore the experiences, perceptions and understanding of albinism from the perspective of mothers of children with albinism in Malawi’.	Qualitative design	Mothers of children with albinism	Individual interviews	Relatives and family members were disappointed, as they were the first ones to have children with albinism. The respective families of albinism children were mocked. Raising a child with albinism in the community was not easy because of beliefs, myths, social isolation and discrimination, mockery and the fear of your child being killed or attacked.
3	Machoko (2013) Zimbabwe	‘To examine albinism in Zimbabwe within its historical context’. ‘To explore the killing of people with albinism at birth’. ‘To explore the role played by Christianity in trying to stop the infanticide of people with albinism’.	Qualitative design	People with albinism Mothers and fathers who gave birth to children with albinism. Chiefs Member of Zimbabwean National Traditional Healers Association and Zimbabwe Traditional Medical Practitioners Council	Individual interviews	Occult beliefs and practices were fuelled by negative attitudes toward people with albinism. In the Mwari cult, people with albinism were seen as spiritual intermediaries, credited with the power to incite political change, control rainfall and protect the natural world. People with albinism were forbidden from marriage, as they were believed to be mermaids – destined not for wedlock, but for ritual intercourse by chiefs and their kin, thought to strengthen chieftainship or bring prosperity to their families. Harmful myths claimed that using the body parts, engaging in ritual sex with or collecting the sperm of a person with albinism could cure illnesses like cancer and HIV/AIDS, while also granting financial success. Herbal concoctions made of body parts of a person with albinism were used as a way of getting powers of the water spirits, the healing, financial and material blessings which were in the body parts of a person with albinism. People living with albinism are seen as suitable ritual sacrifices for one to accumulate wealth. Because of the beliefs that people with albinism were water gods who were emissaries from the rivers, pools of water, dams, lakes and oceans with lots of blessings and healing powers within their bodies.
4	Masanja et al. (2020a) Tanzania	‘To assess the attitudes towards albinism and people living with albinism’.	Quantitative design	Households living with people with albinism	Structured Questionnaires	People with albinism were not seen as part of the community. Communities condemn people with albinism based on their condition rather than appreciating their humanity. The community thought that children born with albinism carried bad luck.
5	Masanja et al. (2020b) Tanzania	‘To explore and understand more about beliefs and practices related to people with albinism’.	Qualitative design	Key informants	Interviews	Children born with albinism are not celebrated. Children with albinism are denied access to education, as they face numerous obstacles in school. Children are mostly attacked because it is easy to get them. The body parts of people with albinism are said to be a powerful medicine for prosperity.
6	Tambala- Kaliati, Adomako and Frimpong (2021) Malawi	‘To examine the problems faced by people with albinism in Malawi’.	Qualitative design	People with albinism: Key informants	Focus groups	Traditional beliefs about albinism compel mothers to keep their children at home. Because of a lack of knowledge and reliable facts about albinism, mothers are mocked by society and their psychosocial well–being suffers. When a child is born with albinism, their name is dependent on the family’s situation at that particular moment. People with albinism have lost faith in both the family and the community.
7	Taylor et al. (2021) Uganda	‘To explore the reactions to the birth of a baby with albinism in Uganda’.	Qualitative design	Parents of children with albinism, people with albinism, traditional birth attendants, nurses, midwives, teachers	Individual interviews	Reactions from villagers and community members reinforced the sense of fear and rejection. The community members were curious about the birth of a child with albinism. When people heard that my mom gave birth to a child with albinism, they said it was not a human being but something. In the community, after my mother gave birth to me, her friends distanced themselves, and the family stopped visiting or inviting her. There was acceptance of a child born with albinism, as they had previous experiences. The general reaction to the birth was one of blame and accusation as to how the baby was different in appearance from the rest of the community. The initial reactions to the birth of a child with albinism were negative.
8	Diale (2021) South Africa	‘To investigate the misguided beliefs and subcultural myths around the birth, nurturing and caring of children with Albinism’. ‘To understand the knowledge and perceptions of the township dwellers on the issues around albinism’.	Qualitative design	People with albinism, the community.	Individual interviews	People with albinism do not die, but disappear. They know that they are a curse to the family.
9	Veldman (2019) Tanzania	‘To assess and compare two radio education strategies about albinism among community members in Kigoma, Tanzania, aiming to improve their knowledge about albinism and reduce stigmatising attitudes towards people with albinism’.	Mixed-methods	Community members	Interviews Surveys	Community felt scared when they saw children with albinism. The community felt pity for them. They thought albinism was caused by ghosts. They thought albinism was contagious. The community thought that parents did something wrong, and therefore God was punishing them.

*Source:* Adapted from Souza, M.T.D., Silva, M.D.D. & Carvalho, R.D., 2010, ‘Integrative review: What is it? How to do it?’, *Einstein (São Paulo)* 8(1), 102–106. https://doi.org/10.1590/s1679-45082010rw1134

Note: Please see full reference list of this article: Mokwele, T., Shirley Mooa, R. & Sepeng, N.V., 2025, ‘Community perceptions towards children living with albinism in Africa: An integrative review’, *African Journal of Disability* 14(0), a1718. https://doi.org/10.4102/ajod.v14i0.1718 for more information.

No., number.

Data analysis is the process of consolidating separate data points by reducing them into a coherent statement about the research problem (Russell 2005). This process involved extracting relevant findings from the reports using a standardised data extraction tool (Oermann & Knafl 2021). The extracted data were systematically analysed to uncover recurring themes by using thematic analysis to identify trends across the study, following Braun and Clark’s (2021) six steps for thematic analysis.

### Ethical considerations

This review is part of a doctoral study aimed at developing community-based interventions for caregivers of children with albinism. Ethical clearance was granted by the University of Pretoria Research Ethics Committee (reference number: 444/2024); however, human participants were not included in the review.

## Review Findings

The results of this review are presented in a narrative format. The review resulted in two main themes: cultural and social perceptions. Table 2 illustrates the themes and sub-themes prevalent in the literature evaluation. The following is an outline of each of these themes and their corresponding sub-themes.

**TABLE 2:** Outline of identified themes and sub-themes.

Themes	Sub-themes
1. Cultural perceptions	1.1 Cultural beliefs 1.2 Mythology and superstition
2. Social perceptions	2.1 Discrimination and stigma 2.2 Lack of knowledge

### Theme 1: Cultural perceptions

Cultural underpinnings shape the way a community defines, understands and perceives children with albinism within their cultural context. The cultural underpinnings are described below: cultural beliefs, myths and superstitions and violence.

#### Sub-theme 1.1: Cultural belief

Cultural beliefs surrounding albinism are complex and often contradictory, indicating how people fear and revere albinism. They often regard people with albinism as spirit mediums (Machoko 2013). Other communities perceive people with albinism as not fully human, believing that they do not die but disappear (Diale 2021), or that they are spirit mediums who are connected to powerful forces like the Mwari cult, able to cause the rain to fall (Machoko 2013). Some perceive them as aquatic beings, ‘mermaids’ or ‘water gods’, possessing healing powers (Machoko 2013).

Owing to cultural beliefs, a child born with albinism is named depending on or according to the family situation (Tambala-Kaliati et al. 2021). Therefore, for children with albinism, they may be named ‘Mabvuto’, which means ‘Trouble’ as a way of expecting what is to come. Communities ostracise and see them as a curse, which demonstrates the tension between cultural beliefs and the reality that people with albinism face. Furthermore, they are forbidden from being married (Machoko 2013) and are instead used for ritualistic sexual practices for the benefit of chiefly lineages. This belief disregards their existence as human beings and indicates that their birth is largely dictated by shame or misery.

#### Sub-theme 1.2: Mythology and superstitions

Communities have deeply entrenched myths and superstitions surrounding albinism that promote harmful misconceptions and discriminatory practices. Caregivers are mostly blamed for giving birth to a child with albinism (Veldman 2019), which is seen as a punishment from God or the result of being supernaturally impregnated by ghosts (Bradbury-Jones et al. 2018). Albinism is often believed to be contagious (Bradbury-Jones et al. 2018) or a result of witchcraft, and people with albinism are believed to carry bad luck (Masanja et al. 2020a) or are linked to ghosts. These beliefs fuel horrific acts, including the killing of newborns with albinism and the use of their body parts for ritualistic purposes (Machoko 2013, 2020). The belief that their body parts possess medicinal or spiritual powers encourages practices such as ritualistic sexual intercourse for healing, wealth and ancestral blessings (Machoko 2013).

The studies extracted indicated that communities often thought that people with albinism were diamonds and therefore put a price tag on their body parts (Masanja, Imori & Kaudunde 2020b). This perception perpetuates the culture of violence against children with albinism. The birthing attendants report that babies born with albinism are killed at birth and their bodies buried together with water plants along the riverbanks to send their spirits to the water source (Machoko 2013; Masanja et al. 2020a). This violence is driven by myths, indicating a disregard for human life and dignity and thus transforming people with albinism into commodities for exploitation and ritualistic abuse. Such myths and superstitions perpetuate an environment of fear and violence, dehumanising people with albinism and marking them as something other than human.

### Theme 2: Social perceptions

Social perceptions of albinism are significantly shaped by cultural contexts and misconceptions. People’s perceptions lead to stigma, as albinism is associated with witchcraft, superstition or a curse. Because of a lack of knowledge and understanding, the discrimination and stigma that people with albinism face leads to social isolation and, therefore, foster fear and prejudice against them. Therefore, discrimination, stigma and lack of knowledge are outlined below.

#### Sub-theme 2.1: Discrimination and stigma

The lives of people with albinism are mostly impacted by persistent stigma and discrimination within their communities. Fear, misinformation and deeply rooted myths foster an environment of social exclusion, where children born with albinism are not celebrated but rather feared and pitied (Bradbury et al. 2018; Masanja et al. 2020b; Veldman 2019). The general reaction to the birth of a child with albinism is one of negativity, blame, fear and disappointment, highlighting a deep-seated prejudice that prioritises difference over appreciating humanity (Likumbo et al. 2021; Masanja et al. 2020a; Taylor et al. 2021). Therefore, families are often mocked and socially isolated, with mothers bearing the blame and suffering from psychosocial distress (Likumbo et al. 2021; Tambala-Kaliati et al. 2021; Taylor et al. 2021).

Children are denied educational opportunities, kept at home because of fear of being abducted and subjected to constant scrutiny and negative reactions (Likumbo 2021; Masanja et al. 2020b; Tambala et al. 2021). Veldman (2019) reported that this negative reaction causes psychological stress and isolation; however, even after educational interventions, communities continue to stigmatise people with albinism (Veldman 2019). Schoolchildren and teachers have misconceptions about albinism being contagious, leading to calls for children with albinism to be segregated.

#### Sub-theme 2.2: Lack of knowledge

A lack of knowledge about albinism within communities influences harmful misconceptions and discriminatory practices. This ignorance manifests in various ways, from communities having different perceptions of what causes albinism; for example, previously, communities thought that ghosts caused albinism (Baker et al. 2021). This thinking caused them to be fearful when they saw a child with albinism, leading them to think that albinism is contagious and to seek children to be taught in different classes (Bradbury et al. 2018; Veldman 2019). Because of a lack of understanding from communities they reside in and the schools they attend, children with albinism often feel lonely and lack a sense of belonging (Likumbo et al. 2021). Therefore, it can be concluded that deep-seated cultural beliefs act as barriers to accurate information, hindering a clear comprehension of the condition.

## Discussion

This review aimed to synthesise the existing literature on community perceptions of children with albinism. The review findings indicated that community perceptions are culturally informed; therefore, cultural beliefs, mythology and superstitions, discrimination and stigma and lack of knowledge will be examined.

The studies included in this review were conducted in Africa; therefore, African cultural beliefs play an important role in shaping their thinking and how they see the world around them (Ned 2022). Lukalo and Maseno (2025) found that Africans have a negative view of disability; as a result, a child born with a disability is seen as a curse. This view is supported by Imafidon (2017), who identified that African ontology isolates those with unusual natures and therefore treats them differently. Therefore, this view provides evidence of the complexities within which children born with disabilities find themselves. The belief that people with albinism have superpowers indicates the cultural significance attributed to their existence by communities, as it suggests that they possess unique powers. This perspective may seem positive; however, Imafidon (2017) expressed that it poses a threat as it reduces their being to their ‘sacredness’ and not being accepted and appreciated as human beings who have their own identities. These views often lead to people with albinism being marginalised out of fear by communities.

In African culture, names given to children are influenced by the family’s circumstances, including albinism. By framing their birth in terms of cultural stereotypes and shame, communities reinforce harmful stereotypes (Moasun & Mfoafo-M’Carthy 2021), making it difficult for them to be accepted by communities. This was supported by Steyn (2022:13), who sought to examine the reasons and causes of prejudice and the consequences of negative designations that stigmatise and victimise people with a form of albinism. The study explained that derogatory nicknames were used to define skin colour such as being called ‘Ghost’ or ‘Inkawo’ in Isixhosa. This can lead to psychological and social implications for people with albinism, leading to isolation. Cultural beliefs and practices against children with albinism violate their human rights; therefore, addressing these beliefs is important to ensure an inclusive environment for them.

Myths influence perceptions and, ultimately, how children with albinism are treated in different cultures. In certain communities, albinism is viewed through the lens of mythical beliefs, where people with albinism are seen as spirit mediums, possessing a level of spiritual power. Daklo and Obadire (2024) acknowledged that, though myths may cause the community to have a sense of reverence towards people with albinism as they are seen as gods assigning a ‘higher’ level of social standing to children with albinism, they simultaneously reinforce their marginalisation.

Superstitions also contribute to the growing misconceptions, as communities believe that people with albinism do not die. According to Aborisade (2021), the belief that children with albinism possess magical powers can lead to violence and, at times, death, as they are easy targets (Mswela 2017). Kajuri and Nyimbi (2019) explained that the belief that people with albinism can bring wealth and health is solely dependent on a person with albinism being mutilated or being sexually violated to cure the perpetrator from an ailment. This indicates a deep-rooted problem and the acceptability of violence motivated by cultural beliefs.

The people with albinism report by Ero et al. (2021) found that children with albinism are discriminated against and stigmatised outside and inside of school, creating a barrier to their development, social integration and well-being. Similarly, this was the case in a study by Brocco (2015), who found that they experience verbal abuse, whereby they are called names such as ‘mwari’ and ‘lekgowa’ and other derogatory names. These often increase the chances of bullying children with albinism. Ringson (2018:13) found that discrimination and stigmatisation take place in communities as there is no cohesion and therefore recommended the creation of a community-based protection model to protect children with albinism.

The right to accessible and inclusive education for children with albinism, as stipulated in the Sustainable Development Goals (SDG 2030), Goal 4, is violated by schools, as they are not willing to teach children with albinism in mainstream schools. Hammond (2020) found that teachers’ unwillingness to integrate children with albinism into mainstream education perpetuates a cycle of exclusion and indicates a systematic barrier they encounter. This was supported by Dapi et al. (2018) and Daklo and Obadire (2024), who further identified that discrimination and the stigmatisation of children with albinism, reflect the broader societal attitudes that marginalise them and foster feelings of inferiority, leading to bullying and negatively impacting their mental health. However, a thematic analysis by Alizadeh et al. (2024), which focused on psychological experiences and support resources for people with albinism, found that teachers served as a source of support for students with albinism. Although this view contradicts prior studies (Daklo & Obadire 2024; Dapi et al. 2018; Hammond 2020), it indicates that when teachers are well capacitated, they can support students with albinism and foster an inclusive environment.

A study by Kajuri and Nyimbi (2020) aimed at describing the impact of myths and superstitions for people with albinism in Tanzania found that the lack of sound knowledge, an information gap allowed misinformation, myths, superstitions and prejudice to thrive. This was supported by Imafidon (2022), who reiterated that lack of knowledge is created by different factors such as lack of epistemic access, the tendency to hold on to beliefs and the quest to defend and sustain them. Kajuri and Nyimbi (2020) further recommend that the community be educated on the medical and genetic causes of albinism. As a result of advancements and the availability of information, it can therefore be deduced that the greatest cause of the lack of knowledge is holding on to cultural traditions and ignorance. Stereotyping thrives when communities do not take it upon themselves to learn. The lack of knowledge, along with cultural beliefs related to albinism communities, may lead people to see people with albinism as threats and therefore lead to social exclusion, stigmatisation and even violence.

## Conclusion

This review synthesised the existing literature on the perceptions of communities regarding children with albinism. The findings indicate that African culture and traditions influence how children with albinism are perceived. Cultural perceptions indicate how cultural beliefs, myths and superstitions perpetuate harm and violence towards children with albinism. Societal perceptions perpetuate discrimination, stigma, exclusion and violations of their fundamental human rights. Comprehensive educational initiatives, community outreach, advocacy efforts and the active involvement of community leaders aligned with the SDGs are required to address cultural and societal perceptions. This can be achieved by fostering awareness and understanding, and communities can work towards reducing stigma and discrimination, creating a supportive environment for people with albinism. Future research should focus on developing community-based protection models to ensure that children with albinism are raised in safe environments.

### Limitations and strengths of the review

This review was limited to studies that focused on the perceptions of community members towards children with albinism in Africa. Only studies conducted in English were included; therefore, studies conducted in other languages, but which were relevant, were excluded. The reviewers conducted a comprehensive search of the included databases; however, some studies may have been omitted.
